# Weight control and behavior rehabilitation in a patient suffering from Prader Willi syndrome

**DOI:** 10.1186/s13104-016-1981-y

**Published:** 2016-04-01

**Authors:** Rosaria Di Lorenzo, Sara Sberveglieri, Donatella Marrama, Giulia Landi, Paola Ferri

**Affiliations:** Psychiatrist, SPDC, Department of Mental Health, Az-USL-Modena, Via P. Giardini, 1355 Baggiovara, 41126 Modena, Italy; Registered Nurse, School of Nursing, University of Modena and Reggio Emilia, Via del Pozzo, 71, 41124 Modena, Italy; Psychiatrist, Outpatient Care Service, Department of Mental Health, Az-USL-Modena, Via I. Newton, 150, 41121 Modena, Italy; Resident in Psychiatry, School of Psychiatry, University of Modena and Reggio Emilia, Via del Pozzo, 71, 41124 Modena, Italy; Nurse Researcher, School of Nursing, University of Modena and Reggio Emilia, Via del Pozzo, 71, 41124 Modena, Italy

**Keywords:** Genomic imprinting, Hypothalamic dysfunction, Hyperphagia and obesity, Aggressive behaviour, Long-term individualized rehabilitation

## Abstract

**Background:**

This study reports a case of Prader Willi syndrome (PWS), a genomic imprinting disease related to chromosome regions 15q11.2–q13 15, which includes hypothalamic dysfunction leading to hyperphagia, obesity, shortness, sleep abnormalities. Our case is extremely severe, in comparison to other PWS cases described in literature, due to the association with severe emotional and psychiatric symptoms: oppositional behaviour, rigidity of thought, skin picking and pathological hoarding.

**Case presentation:**

We described the case of a Caucasian male patient suffering from PWS, treated in outpatient care by local Mental Health Centre and supported by Social Service, who was admitted to a residential rehabilitative facility. After a 2-year follow-up, the patient showed a global improvement in symptoms and functioning, as registered by the rating scales administered. At the end of observation period, we also reported an important improvement in weight control, reducing the risk of obesity and related diseases, therefore improving the prognosis of life.

**Conclusion:**

This case highlights the need for long-term, individualized and multi-professional treatment in patients suffering from a complex genetic syndrome with both organic and psychological alterations, for which medical care setting and pharmacological treatments are not sufficient. Clinical observation of this case leads us to compare PWS to drug addiction and indirectly endorse the neurophysiological hypothesis that food and drugs stimulate the same brain circuits in the limbic system.

## Background

### Genetics and diagnosis

Prader-Willi syndrome (PWS), described for the first time in 1956 by the Swiss doctors from which it takes its name, presents a prevalence ranging from 1:10,000 to 1:30,000. It is present in both males and females of all races and is rarely inherited (only one person in a family is usually affected by this syndrome) [1].

PWS, the first human disorder to be recognized as related to genomic imprinting, is caused by the failure of expression of paternal genes presented in the region of chromosome 15q11.2–q13 15 [2, 3]. The main molecular mechanisms responsible for PWS are [4]:

Paternal microdeletion (75–80 % of cases); maternal disomy (UPD) (20–25 % of cases); imprinting defect (ID), (1–3 % of cases); other defects, such as balanced and unbalanced translocations, which with ID are responsible for most cases of familial inherited PWS.

A set of clinical criteria was proposed by Gunay-Aygun and collaborators [5] in order to identify the subjects which required an appropriate genetic test to diagnose PWS: poor muscle tone, reduced ability to suck milk and, if male, cryptorchidism in early childhood and delay in neurological developmental, short stature, psychomotor retardation, obsessive behaviour, compulsive ingestion of food with obesity in middle childhood and adolescence.

PWS diagnosis requires the execution of a test called DNA methylation (specific multiplex ligation-dependent probe amplification analysis, abbreviated MS-MLPA).

### Endocrinological, cognitive and behavioural alterations

Most endocrine disorders in PSW are secondary to hypothalamic dysfunction [6], which is responsible for the deficiency of growth hormone, thyroid stimulating hormone, adrenal-corticoid hormones and hypogonadism [4, 7]. The reduced secretion of gonadotropins and growth hormone condition neonatal hypotonia, short stature, puberty failure, as well as all the behavioural manifestations that are associated with this syndrome. Central hypothyroidism, with a normal value of thyroid stimulating hormone and low levels of free thyroxine, has been documented in approximately 25 % of individuals with PWS [1]. Other key symptoms, such as hyperphagia and altered control of appetite [8], alteration of sleep-wake cycle [6], dysfunction of body temperature regulation [9] and increased pain threshold [10] would be secondary to alterations in hypothalamic function [8]. Obesity in PWS is mainly central (abdomen, buttocks and thighs) in both sexes, with a reduced amount of visceral fat compared to other patients with the same degree of overweight [11]. Several studies have shown that ghrelin levels are significantly elevated before and after meals in hyperphagic individuals with PWS, especially in older children and in adults. In children with PWS has been found hyper-ghrelinemy [12], but this result has not been replicated in other studies [13, 14]. Some authors have examined the neural circuit that mediates appetite and eating behaviour (orbitofrontal cortex and hypothalamus) and have shown, through functional magnetic resonance imaging, that this circuit in PWS is abnormally activated during the intake of high-caloric food [15, 16]. Reduced sleep latency rapid eye movement (REM) with altered architecture of the different stages of sleep, oxygen desaturation and obstructive apnea have been reported in PWS [17].

A literature review of cognitive abilities among 575 individuals affected by PWS showed that only 5 % of these patients had a normal IQ [4]. 70–90 % of patients with PWS present behavioural disorders since childhood, characterized by impaired impulse control with bursts of anger, manipulative attitudes [4], and compulsions, in particular pathological hoarding (especially food) and skin picking [18, 19]. Some authors have observed that many of these behavioural alterations are also present in autism spectrum disorders and that both the disorders may be associated with genetic alterations of chromosome 15 [20]. Forster and Gourash [21] have identified five domains related to behavioural alterations observed in patients with PWS: hyperphagia and related behaviour, hostility, rigidity of thought, skin picking and pathological hoarding, feelings of insecurity with increased anxiety. In a Japanese study, the authors have highlighted that patients with PWS often present anxiety disorder, obsessive compulsive disorder and mood instability in comorbidity [22].

Complications are almost all related to the underlying endocrine alterations: osteoporosis with fractures, secondary hypogonadism disorders, sleep apnea, cardiovascular disease, diabetes mellitus type 2 and all complications related to the obesity that develops in almost all cases. Other complications are related to the frequent binges that can result in death due to self-suffocation, rupture of stomach and, in the case of ingestion of inedible substances, poisoning. The behavioural alterations can further complicate the course of psychological development and lead to a serious socio-environmental maladjustment [23].

The only drug therapy indicated for this syndrome is growth hormone, which was approved in 2000 by the Food and Drug Administration (FDA) for the treatment of PWS in childhood [4]. This pharmacological treatment significantly reduces the initial muscular hypotonia and the subsequent risk for the development of obesity, partially normalizes physical and psychological development and improves cognitive function. The prescription of this drug in adulthood is still debated due to the possible aggravation of diabetes and sleep apnea and the increased risk for edema and cancers [7, 11]. Many authors have identified multidisciplinary rehabilitative programs as indispensable tools to treat these patients [21, 24]. To support families and caregivers of these patients, many voluntary associations have been organized. In Italy, on 12 July 1991, sixteen families living in Lombardy founded the first association for people with Prader Willi (www.praderwilli.it) and, subsequently, other associations were founded [4].

### Purpose

We describe the case of a male Patient suffering from PWS, treated in outpatient care by Mental Health Centre (MHC) and supported by Social Service since childhood, who was admitted to a residential rehabilitative facility (RRF), in April 2012. This RRF is dedicated to people with severe and very severe mental disabilities. After collecting anamnestic information of the Patient from the medical records of out- and inpatient psychiatric services and from the reports of parents and social assistants respectively, we analysed the rehabilitative program developed for the Patient in the RRF as reported in the documentation and the clinical chart of this institute. In order to assess the multi-professional clinical outcome of 24-month rehabilitative program at the RRF, from April 2012 to 2014, we evaluated, at the end of the observation period, the number of psychiatric admissions, hematologic tests and body mass index (BMI), behavioural alterations and functioning levels by administering symptom and functioning rating scales: the Italian scale, so called “VADO”: Valutazione di Abilità, Definizione di Obiettivi (evaluation of ability, definition of objectives) [25], Personal and Social Performance Scale (PSP) [26]. Finally, we report the current state of the Patient, updated in June 2015, in order to better detail the outcome of his rehabilitation program.

## Case presentation

### Childhood

At the age of two, the Patient was abandoned by his parents as were his brother and sister. Successively, he and his siblings were adopted by Italian parents. In the new family, our Patient lived in a detached country house with four of his adoptive brothers and two young children, sons of two adoptive brothers, presenting good relationship with all components. Shortly, in early childhood, he presented severe hyperphagia and compulsive search for food and inedible substances, developing a sort of Pica. His adoptive mother referred that he was so attracted by substances with a good smell to eat scented soaps and drink bubble baths. Also, disgusting things such as rabbit faeces or rotten rubbish were stolen and eaten by him. When he was 3 years old, he was short but already weighed 33 kg. In the meantime, he progressively developed aggressive behaviour, especially in case of prohibitions or restriction of food. At age 4, he was diagnosed with PWS by the methylation test in chromosomal region 15q11–13, which revealed the presence of a single methylated band, whose molecular evidence has been associated with the most common PWS phenotype. From that point onwards, his family implemented new restrictive rules, such as to lock the pantry or to lock him in his room during the night, in order to prevent the patient gaining weight or swallowing something inedible. Moreover, the patient’s family did not buy caloric food or scented substances and put in place many distracting activities for the Patient (watching movies, completing puzzles, etc.). His mother reported that, on more than one occasion, in order to prevent poisoning, the family consulted Emergency because the Patient had ingested leaves or other vegetable products with unknown effects. From kindergarten, his attendance at school was very problematic, despite the support of teachers dedicated to him, due to many behavioural alterations, learning difficulties, inability to maintain attention, aggressiveness, intolerance to frustration, lack of socialization skills and continuous search for food. He completed middle school, but, due to his problems, was rejected by more than one high school. Finally, he enrolled in a vocational school, where he attended only the first year, which he had to repeat, before dropping out. Despite his school failure, the Patient was able to cultivate some interests such as reading, drawing and composition of puzzles. In particular, he showed great skill in performing puzzles composed of many pieces. In addition, he was really interested in horror movies and comics, which could sometimes distract him from the compulsive search for food. When he was 17 years old, the Patient ran away from home, after being discovered by his parents in the act of eating a rotten cake collected from the garbage. Readily found by the Police, he claimed not to want to return to his family, complaining about abuses suffered in his family home (“My parents lock me in my room”). Therefore, the local Social Service for Minors, promptly activated, provided for the temporary placement of the Patient in a community. In the meantime, the Patient was admitted to the psychiatric day hospital of an accredited private hospital dedicated to adolescent psychiatric patients, in order to evaluate his psychological profile (interpersonal skills, reaction to stress and tolerance for frustration) and assess, in the meantime, the ability of the family to adequately support him.

### The first psychiatric hospitalization

After about 20 days of staying in community, our Patient presented such a dangerous aggressive behaviour against another boy who lived in the community, as to be hospitalized in a psychiatric ward. He was discharged after a few days, and sent to the psychiatric ward dedicated to adolescent patients in a private hospital, where he remained hospitalized for about 3 months. During this hospitalization, many clinical features of PWS emerged, in particular hyperphagia and severe behavioural alterations, characterized by outbursts of anger and violence toward objects and people. WAIS was administered to him, where he obtained a score of 68 on verbal IQ scale and 62 on performance IQ scale On the Vineland scale, the patient showed a result consistent with mild mental impairment.

### The first rehabilitative program

At discharge from the private psychiatric hospital, he was sent back to his adoptive family with a rehabilitation program, which provided for daily attendance at the rehabilitative centre of a mental disability facility. Clinical conditions of the Patient remained stable for 5 months. Afterwards, he was again hospitalized in the public acute psychiatric ward due to agitation and aggressiveness, apparently motivated by frustration for being scolded by both professionals and parents for his “sentimental effusion” towards a female patient who attended the same rehabilitative centre. After few days, he was discharged and sent to the adult outpatient psychiatric services since he had come of age.

### The long psychiatric hospitalization

A few days after previous hospitalization, he was re-admitted to the same acute psychiatric ward, due to an outburst crisis at the rehabilitative centre, reactive to the attempts of professionals to control his dangerous abuse of water. He binged daily on water, especially at lunch time, in response to rigid reduction of food intake. During this hospitalization, the Patient presented dangerous behaviour: abuse of water, theft of food from other patients (also leftovers from dishes or rubbish bins) and theft of money to buy food. In particular, he remained awake at night to go hunting for food and money in the rooms of other patients. The Patient also used to hoard stolen food in some hiding places on the ward (including in the toilet bowl), where he went to eat food unseen. When he was discovered committing these acts as well as when he was prohibited eating more than his regulated diet, he became extremely aggressive with explosive and unstoppable violence against people and objects surrounding him. It was necessary to pharmacologically and physically restrain him repeatedly. During this long hospitalization, mother, who, in the meantime, was nominated his legal guardian, referred not to be available to take him home again due to his dangerous behaviour, which she and her husband were not able to contain. She asked to place the Patient in a rehabilitative facility in order to modify his behaviour. After a month of hospitalization, the Patient presented aggressiveness frequently difficult to contain as well as uncontrollable food intake (he had gained about 10 kg since admission), although he continued his daily rehabilitative activities in an outpatient service. Therefore, a multi-professional meeting planned to transfer the Patient to a residential rehabilitative facility (RRF) for people with disabilities, and to dedicate professionals to him 24 h a day, at least in the beginning of the program.

### Rehabilitation in the residential facility

At admission to the RFF, a complex and intensive program which provided maximum control over food intake and, at the same time, various physical activities and recreations, was tailored for the Patient.

A. Control of food intake. A low-fat diet with MCT (medium-chain triglyceride) of 1700 kcal/day, divided into three main meals and two snacks, one in the morning and one in the afternoon, was provided; the Patient was not allowed to consume morning and afternoon snacks with other patients, having a dietary restriction that others did not have and other many prohibitions related to food were established (no access to the kitchen pantry day or night, no change to the timing of meals in order to maintain regular life habits, no using food as bargain or as reward or punishment in order to reduce positive reinforcement by food).
B. Motor activities. An intensive program was carried out, under the constant supervision of educators, which included physical activities and games: gymnastics with a frequency of four times a week at the RRF gym, soccer play with the other guests and swimming in the pool once a week; physical activity under teacher supervision at a gym once a week. During the rest of the time, the Patient was encouraged to walk as much as possible by the operator dedicated to him in order to counteract the tendency to weight gain and, at the same time, to distract him from the compulsive search for food.
C. Activities aimed at improving socialization and autonomy capacity. Many organized recreational activities were planned: visits to farms, libraries, playgrounds, leisure centres, etc., together with other patients who lived in the RRF and with professionals dedicated to him. Regarding money management, educators received money from Patient’s mother, his legal guardian, and delivered money to him, according to some clear rules: he could receive a certain sum to buy only one item during the weekly recreational walk (food purchase was not allowed). In this regard, the Patient had to previously agree with the educator on the item to buy, through a written agreement signed by both parties, and could not change his choice.

During his stay at the residential facility, the patient also continued to be seen regularly by his referring psychiatrist. In these meetings, the Patient revealed his feelings of inadequacy, anxiety, anger and fears. He complained to be suffering due to “the insatiable desire for food” and “attraction towards girls”. All visits ended with the same incessant requests to be admitted to the public psychiatric hospital, where the patient felt “less controlled”. A new psychotherapy for the Patient was carried out by a psychologist from the Prader-Willi Association, focusing on the Patient’s feelings of abandonment by his adoptive mother and his difficulties with others. The Patient was also monitored monthly by the general physician of the RRF, who regularly checked his physical condition (body weight, hematic tests). He was periodically visited by endocrine and nutrition specialists.

### The assessment of outcomes

#### The number of psychiatric hospitalizations during the observation period

**Fig. 1:**
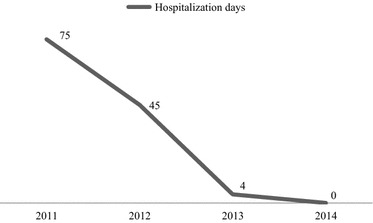
Days of psychiatric hospitalizations during the observation period

After 3 months, the Patient was again admitted to the public psychiatric hospital because of his hostile and threatening behaviour. After his admission, he immediately appeared sufficiently quiet and cooperative as to be promptly discharged and sent back to the RRF in order to continue his program. After another 6 months, he presented a new crisis for the “loss” of his adoptive family requiring compulsory psychiatric hospitalization, due to uncontrollable outbursts of anger and violence. After 7 days, he was discharged to resume his rehabilitative program. Successively, in concomitance with the summer holidays of his referring professionals, momently substituted by other ones, and the reduction of his recreational activities, he presented a progressive increase of dangerous behaviour. In that period, he had physically assaulted a professional, causing him an arm fracture; he had removed the cannula for parenteral nutrition from the body of another patient, in a vain attempt to feed by it; he had repeatedly ingested earth and grass gathered from the garden of the institute and so on. Finally, the Patient was admitted again to the public psychiatric ward, where he presented violence against himself, motivated by the frustration of not being able to eat freely despite his insatiable hunger. In the ward, he was isolated from other patients and controlled 24 h a day by one of the professionals dedicated to him. This intensive control permitted his rapid tranquillization and, consequently, his discharge after few days. He was again sent to the RRF, where he continued his program with new activities: he began a protected work project in an outside rehabilitative centre for 2 h once a week. At this centre, the Patient performed small manual tasks such as assembling and packaging items, under the constant supervision of his professionals, and earned a monthly salary. In the meantime, his adoptive mother reduced her visits to him to no more than once a month hoping to reduce the patient’s tantrums, whereas his biological sister, who lived in the same adoptive family, progressively increased her visits, accompanying him out for walks and home visits. The Patient, who looked forward to going back to his family, presented appropriate behaviour during his monthly authorized home visit, reporting great satisfaction. Successively, he was not hospitalized, showing a progressive improvement in his behaviour with good adaptation to the RRF. As shown in Fig. 1, the days of hospitalizations were significantly reduced to zero at the end of the observation period, fulfilling one of the main outcomes of his rehabilitation program.

#### Pharmacological therapies

The patient was treated with neuroleptics and benzodiazepines due to his severe behavioural alterations, despite the risk for increased appetite. At the initial admission to the RRF, he was treated with 30 mg of aripiprazole and 1500 mg of valproic acid, but after a few weeks, valproic acid was suspended due to its inefficacy to control aggressiveness. During the observation period, the patient frequently required additional sedative therapies for his severe behavioural alterations, like chlorpromazine (100 mg/day) and benzodiazepines (alprazolam 3.5 mg/day, clordemetildiazepam 2mg/day, lorazepam 4 mg/day). The patient had never been prescribed hormonal therapy (growth hormone or testosterone), which was not considered useful by endocrinologists who examined him, given the risk of worsening his behavioural problems.

#### The scale scores

**Fig. 2:**
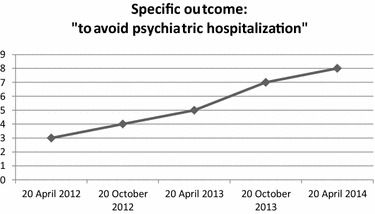
“VADO”: Specific outcome

**Fig. 3:**
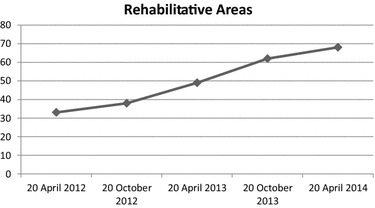
“VADO”: Rehabilitation Areas

**Fig. 4:**
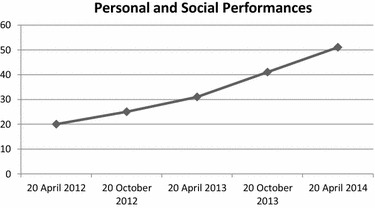
“VADO”: Personal and Social Performances

“VADO”: (a) Specific outcome (Fig. 2), which consisted of avoiding psychiatric hospitalizations, pathologically required by the Patient, was achieved an improvement rate of 63 %; (b) Rehabilitation Areas (Fig. 3), which evaluated the Patient’s adherence to different rehabilitation activities, showed an improvement rate of 51 %; (c) Personal and Social Performances (Fig. 4), which evaluated the Patient’s ability to take care of his personal hygiene and clothing, showed an improvement of 61 %.

**Fig. 5:**
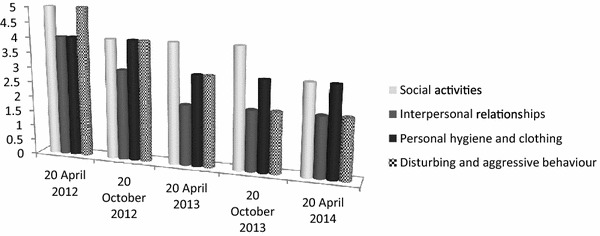
The main areas of Personal and Social Performance (PSP) scale

“Personal and Social Performance Scale” (PSP) reported an overall improvement of 44 % at the end of the observation period, with the highest percentage of improvement (60 %) in his disturbing and aggressive behaviour (Fig. 5).

#### Body mass index (BMI)

**Fig. 6:**
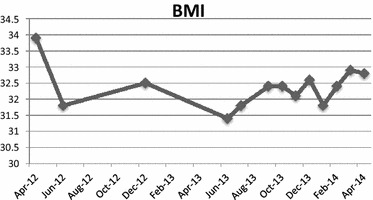
Body mass index (BMI) during the observation period

At the initial admission to the RRF, the patient presented a BMI of 33.9 (weight 90 kg, height of 1.63 cm), higher than the normal value of 24.9. At the end of the observation period, he had a BMI of 32.8, despite the strictly controlled diet and regular physical activity (Fig. 6).

During all internist visits, any significant organic complications were registered; in particular any sign of hypothyroidism or diabetes mellitus was noted. All his hematic tests were mostly normal.

### Current state update

To date, the patient continues his rehabilitation activities at the RRF, with good cooperation despite some moments of anger and aggression that his professionals are able to contain without resorting to psychiatric hospitalization. He follows his usual hypocaloric diet with no extras and, at the last control, he weighed 87 kg (BMI = 32.74) with normal hematic test. Ultimately, he shows an increased interest in hoarding different non-food items such as toy soldiers, comics, horror movies, crossword puzzles, etc., all activities which are allowed in the RRF, suggesting that he maintains compulsive behaviour although directed on different objects from food.

## Conclusion

In our PWS case, psychiatric intervention was necessary because of his severe mental and behavioural alterations: aggressiveness, mental retardation, outbursts of anger, disruptive crisis, obsessive and compulsive behaviour. These symptoms, related to the genetic disorder, had probably been exacerbated by early abandonment by his biological parents and, successively, by the difficulty his adoptive family had in caring him. In line with most studies [2, 27, 28], our case shows that a genetic syndrome can impair individual’s essential needs, leading to severe maladjustment. This condition requires a bio-psycho-social approach in order to take care of both organic conditions and psychological needs without neglecting the environmental conditions.

The psychiatric intervention was closely connected with well-structured rehabilitation programs at the RFF, which stimulated Patient’s capabilities, despite his initial poor self-management skills, and, in the meantime, controlled his behaviour by the regular daily presence of one of the three professionals dedicated to him. This continuous and intense rehabilitation program has led to a significant reduction of hospitalizations, allowing the Patient to avoid the behavioural regression induced by frequent and recurrent admissions, at the cost of an increased dependence on institutions. Despite these positive results, the weight control induced only a slight reduction in BMI, maintaining it within the range of moderate obesity at the end of the observation period. Nevertheless, this result obtained through an enormous rehabilitative and educational effort by the RRF staff, represented a good outcome since further weight gain, which is the common final pathway in most PWS, was avoided. The data show that obesity related to genetically induced hyperphagia can be controlled by a regular and long-term rehabilitative approach tailored to behavioural and psychological alterations. At the RRF, body weight control was carried out 24 h a day through the adoption of different strategies aimed at controlling of food intake and, in the meantime, at reinforcing all activities without any oral gratification. At the end of the observation period, the staff registered a significant improvement in the rehabilitation areas of his personal and social functioning, whereas his referring psychiatrist found most relevant the reduction of his disturbing and aggressive behaviour.

Finally, the clinical observation of this case leads us to compare PWS to drug addiction and indirectly endorse the neurophysiological hypothesis that food and drugs stimulate the same brain circuits in the limbic system [15]. In this regard, many symptoms presented by our Patient, such as impulsivity, craving for food, compulsive overeating and obesity support the concept of food addiction, first developed by Gold’s team [29] and successively interpreted as a Reward Deficiency Syndrome by other authors [30], suggesting a commonality between substance and behavioural addiction. Food addiction, which shares with substance abuse and gambling DSM-5 criteria and brain reward networks, is characterized by craving, as well as other addictions, which, in this case, is for food [31]. Other studies have shown that obese populations with greater BMI present changes in reward pathways similar to those observed in drug addiction. In particular, some research has found that, in individuals with higher BMI, dopaminergic receptors are down-regulated [32] and present greater activation in the gustatory cortex and somatosensory brain regions in the presence of food [33]. Other genetic studies have confirmed biological predisposition to overeating and weight gain, indicating that genetic polymorphism of dopamine D2 receptor and leptin receptor genes are involved in obesity and craving for food [34, 35]. PWS could represent an extreme case biologically conditioned, which could help us to better understand the complex pathophysiological mechanisms that underlie both addiction and obesity. Most studies underline that effective treatments for drug addiction and obesity can be different since the modality of relapse is not similar [36]. Some authors have indicated that a pharmacological strategy aimed at improving dopamine functioning could be effective for both diseases, but, up to now, the results are inconclusive. Nevertheless, due to the complexity of addiction and obesity, which can be strongly influenced by environmental factors, behavioural therapeutic programs aimed at improving the capacity of the individual to control on his/her compulsive behaviour by means of positive reinforces can be effective.

Our clinical case, emblematic for its complex therapeutic and rehabilitative needs, required a multidisciplinary staff since many different activities, ranging from control of organic symptoms to educational and rehabilitation programs, were necessary to manage his multiple symptoms. Nevertheless, an empathic relationship, despite Patient’s manipulative and aggressive attitudes, was necessary to motivate and support him in the course of such a difficult treatment. Although the genetic component heavily influenced the course of this disorder, we can highlight that a long-term, well-defined and individualized rehabilitative program can control pathological behaviour, including hyperphagia, and therefore improve the long-term prognosis. In order to implement these programs, emphatic therapeutic relationships are necessary in order to support and motivate patients in adhering to rehabilitative programs, the only available treatment preventing potential regressive evolution.

## Ethics approval and consent to participate

Written informed consent was obtained from the adoptive mother of the Patient, who is his legal guardian, for publication of this case report.

## Abbreviations

PWS: Prader Willi syndrome
UPD: uniparental disomy
ID: imprinting defect
REM: rapid eye movement
IQ: intelligence quotient
WAIS: Wechsler Adult Intelligence Scale
RRF: rehabilitative residential facility
BMI: body mass index
VADO: Valutazione di Abilità, Definizione di Obiettivi (evaluation of abilities, definition of objectives)
PSP: Personal and Social Performance Scale
MHC: Mental Health Centre
MCT: medium-chain triglyceride
